# Supplementary material for: Microplastic pollution at Qilianyu, the largest green sea turtle nesting grounds in the northern South China Sea
Source: PeerJ. 2022 Jun 7;10:e13536. doi: 10.7717/peerj.13536 (PMC9186329; doi:10.7717/peerj.13536)
Supplement: Supplemental Information 1 [file peerj-10-13536-s001.docx]

**Table S1. Quantities of microplastics in sediments from different areas.**

| **Country** | **Study area** | **Microplastic size range (mm)** | **Abundance±SD (pieces·m^-2^)** | **References** |
| --- | --- | --- | --- | --- |
| China | Qilianyu | 0.05-5 | 1353.78 ± 853.68 | In this study |
| China | Hainan Island | 0.05-5 | 2567.38±2937.37 | (Zhang *et al*., 2021) |
| China | Hong Kong | 0.315-5 | 5595 | (Fok and Cheung, 2015) |
| China | Guangdong Province | 0.315-5 | 6675 ± 7021 | (Fok *et al*., 2017) |
| China | Ganquan and Quanfu Islands | 0.02-5 | 1774.75±1534.37 | (Fang *et al*., 2021) |
