# Supplementary material for: Microplastic pollution at Qilianyu, the largest green sea turtle nesting grounds in the northern South China Sea
Source: PeerJ. 2022 Jun 7;10:e13536. doi: 10.7717/peerj.13536 (PMC9186329; doi:10.7717/peerj.13536)
Supplement: Supplemental Information 2 [file peerj-10-13536-s002.doc]

**Table S2. The average microplastic densities at different depths.**

| **The depths (cm)** | **Abundance±SD (thousand pieces·m^-3^)** |
| --- | --- |
| 0–2 | 418.89 ± 270.41 |
| 2–20 | 415.11 ± 301.35 |
| 21–40 | 277.85 ± 140.14 |
| 41–60 | 264.67 ± 200.40 |
