# Supplementary figures and images for: Microplastic pollution at Qilianyu, the largest green sea turtle nesting grounds in the northern South China Sea
Source: PeerJ. 2022 Jun 7;10:e13536. doi: 10.7717/peerj.13536 (PMC9186329; doi:10.7717/peerj.13536)

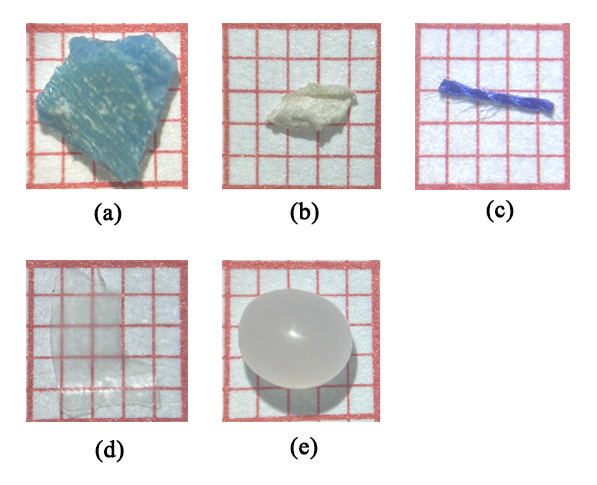

Supplement: Supplemental Information 3 [file peerj-10-13536-s003.jpg]

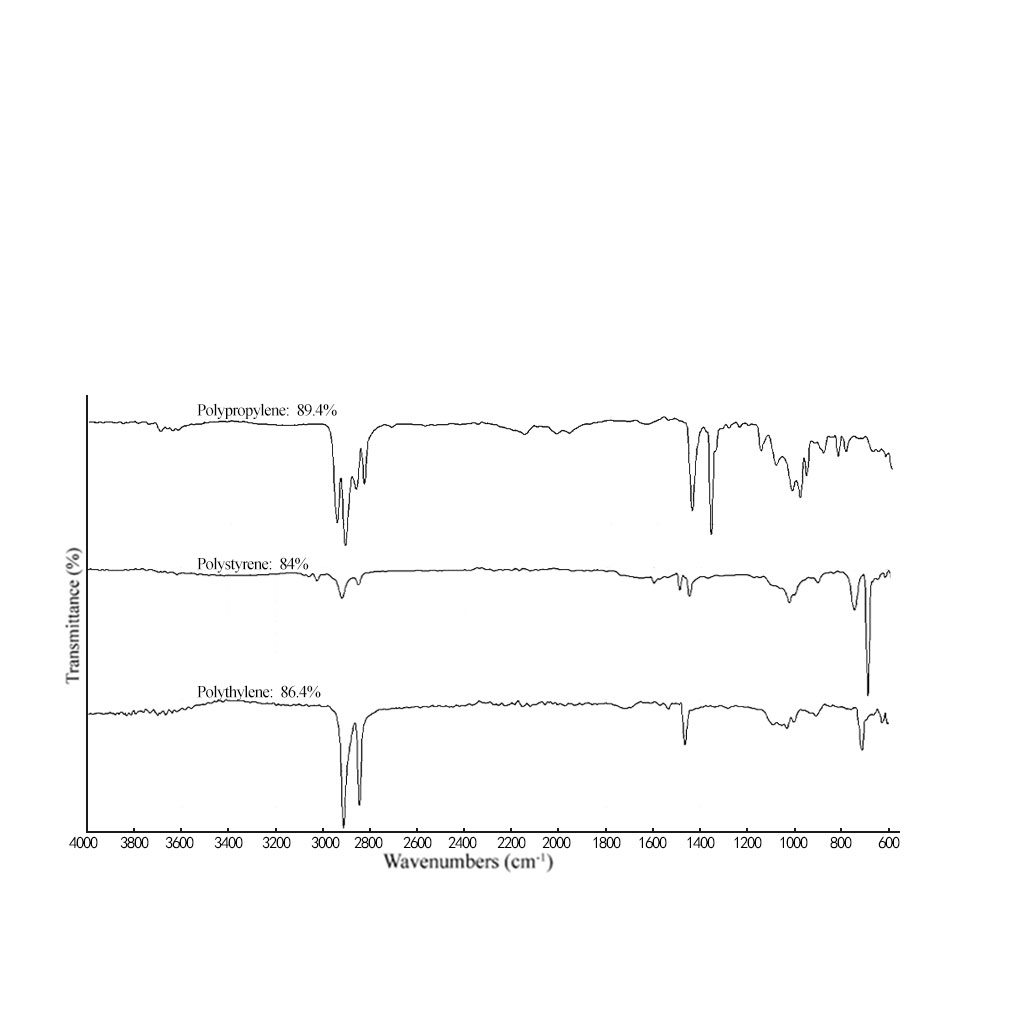

Supplement: Supplemental Information 4 [file peerj-10-13536-s004.jpg]

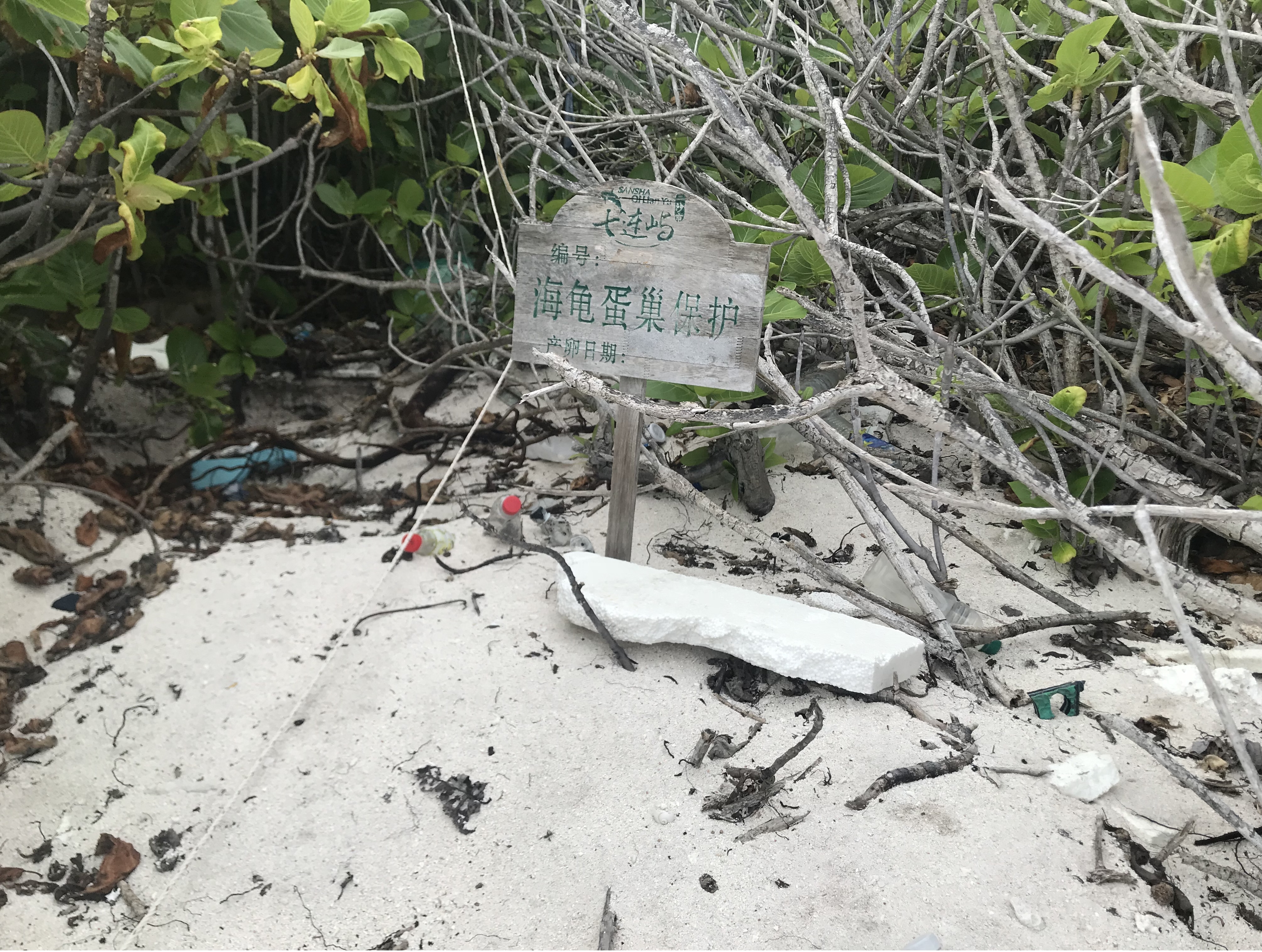

Supplement: Supplemental Information 5 [file peerj-10-13536-s005.jpg]

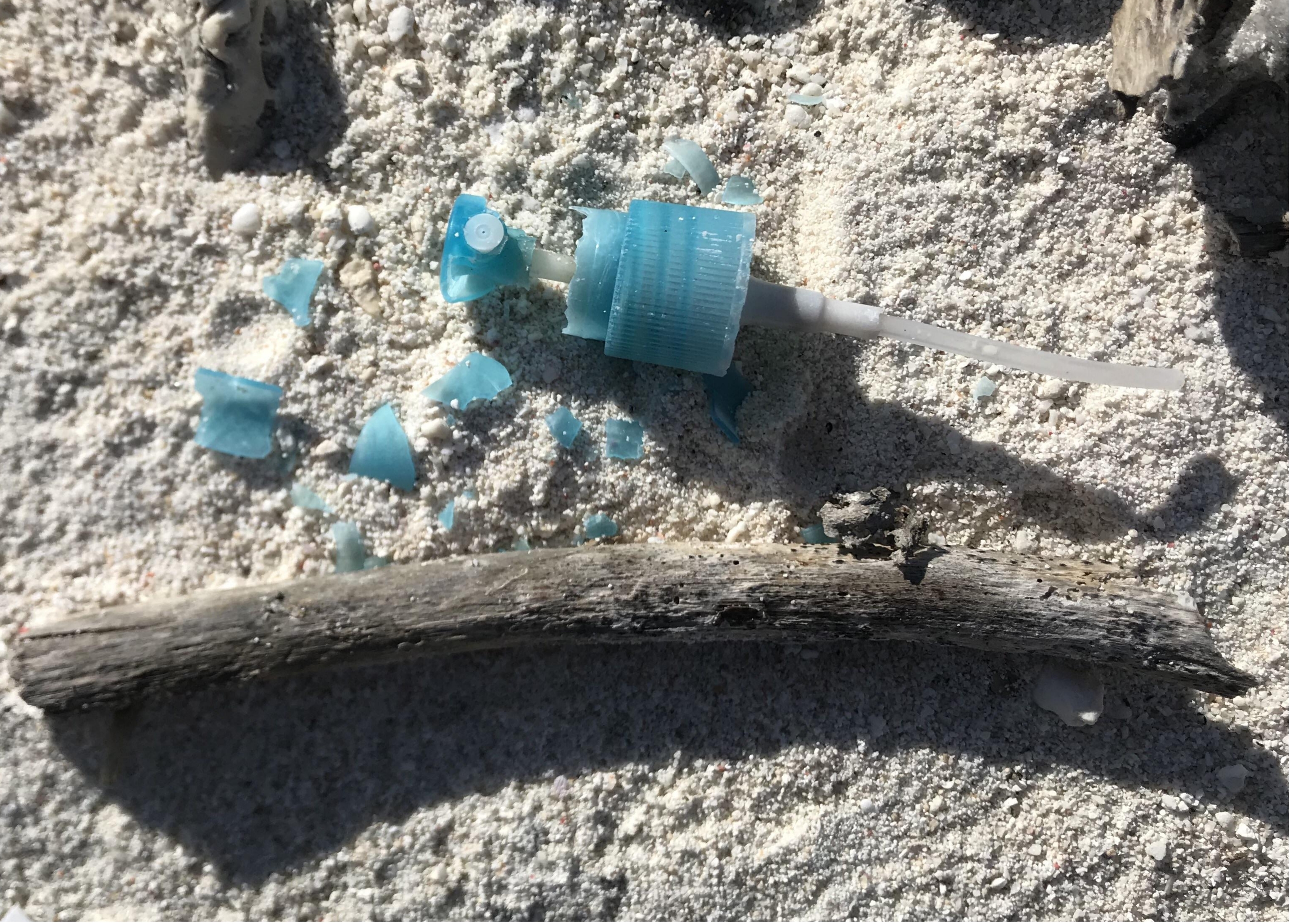

Supplement: Supplemental Information 6 [file peerj-10-13536-s006.jpg]
